# Transcriptome Analysis of Red Swamp Crawfish *Procambarus clarkii* Reveals Genes Involved in Gonadal Development

**DOI:** 10.1371/journal.pone.0105122

**Published:** 2014-08-13

**Authors:** Hucheng Jiang, Zhijun Xing, Wei Lu, Zhaojun Qian, Hongwei Yu, Jiale Li

**Affiliations:** 1 Key Laboratory of Exploration and Utilization of Aquatic Genetic Resources, Shanghai Ocean University, Ministry of Education, Shanghai, China; 2 Jiangsu Xuyi Riverred Crawfish Eco-Park CO. LTD, Xuyi, China; 3 E-Institute of Shanghai Universities, Shanghai Ocean University, Shanghai, China; Temasek Life Sciences Laboratory, Singapore

## Abstract

**Background:**

The red swamp crawfish, *Procambarus clarkii*, has become one of the most economically important cultured species in China. Currently, little is known about the gonadal development of this species. Isolation and characterization of genes are an initial step towards understanding gonadal development of *P. clarkii*.

**Results:**

Using the 454 pyrosequencing technology, we obtained a total of 1,134,993 high quality sequence reads from the crawfish testis and ovary libraries. We aimed to identify different genes with a potential role in gonad development. The assembly formed into 22,652 isotigs, distributed by GO analysis across 55 categories in the three ontologies, ‘molecular function’, ‘cellular component’, and ‘biological processes’. Comparative transcript analysis showed that 1,720 isotigs in the ovary were up-regulated and 2138 isotigs were down-regulated. Several gonad development related genes, such as *vitellogenin*, *cyclin B*, *cyclin-dependent kinases 2*, *Dmc1* and *ubiquitin* were identified. Quantitative real-time PCR verified the expression profiles of 14 differentially expressed genes, and confirmed the reliability of the 454 pyrosequencing.

**Conclusions:**

Our findings provide an archive for future research on gonadal development at a molecular level in *P. clarkii* and other crustacean. This data will be helpful to develop new ideas for artificial regulation of the reproductive process in crawfish aquaculture.

## Introduction

The red swamp crawfish, *Procambarus clarkii*, is a species of freshwater crawfish native to the Southern central United States and Northeastern Mexico [1], and has become one of the most widely introduced crawfish species in the world. This species was introduced from Japan to Nanjing, China, in 1929 [2]. Since the 1990s, *P. clarkii* have been farmed extensively as food source and have become one of the most economically important farmed species in China [3]. Due to its high market demand and high economic value, crawfish aquaculture has developed rapidly in the past decades, and its annual output has reached approximately 479,374 tons, accounting for 91.12% of the global production according to the 2009 United Nations Food and Agriculture Organization report [4]. Owing to the growing culture industry, a shortage of juvenile crawfish has become prevalent [5], leading to severe restriction in large-scale crawfish aquaculture.

With the aim of developing a method for controlling the quality and quantity of crawfish and their eggs in aquaculture, many studies have been investigated on crawfish gonadal development [6]–[8]. Knowledge of mechanisms governing gonadal development processes at the molecular level is crucial and could be directly applied to the crawfish industry. Recently, some genes have been shown as critical factors for gonadal development in crawfish and other crustaceans, including *vasa* [9], *cyclin B* [10], *cell division cycle 2* [11], *elongation factor 2* [12], *heat shock protein 90* [13], *cathepsin C* [14], and *Dmrt* [15]. Generating gonad specific libraries facilitates an understanding of the molecular mechanisms of gonadal development.

DNA sequencing provides important data to study the regulation of gene expression. Although a large number of whole-genome sequencing studies have been performed for microbial [16] and vertebrate [17] in the past decades, crustacean genomes have only been sequenced in *Daphnia pulex* [18]. Transcriptome sequencing provides general representation of almost all transcripts expressed in specific cells or organs at particular conditions and stages. Because of its advantages of high throughput rates and low costs, the 454 pyrosequencing technology is regarded as the first choice for the identification of novel genes in organisms lacking a reference genome [19]. It has thus been employed for other crustacean species, including *Litopenaeus vannamei* [20], *Macrobrachium rosenbergii* [21], *M. nipponense* [22] and *Euphausia superba* [23].

Screening and identifying gonadal differentially expressed genes are an initial step towards understanding gonadal development in *P. clarkii*. In the present study, we performed a 454 pyrosequencing of *P. clarkii* using prepared cDNA from mRNA isolated from testis and ovary tissues. This was used to generate expression profiles and to discover differentially expressed genes in these two tissue types. Based on previous research, and employing bioinformatics tools, we aimed to generate a list of candidate genes that may be involved in the gonadal development of *P. clarkii*. This could provide a major resource for future studies of crawfish gonadal development, and in particular should help to establish new ideas for the artificial regulation of reproductive processes in crawfish aquaculture.

## Materials and Methods

### Ethics statement

The handling of crawfish was conducted in accordance with the guidelines on the care and use of animals for scientific purposes set by the Institutional Animal Care and Use Committee (IACUC) of Shanghai Ocean University, Shanghai, China.

### Crawfish tissue samples collection

The crawfish used in this project were obtained from Jiangsu Xuyi Riverred Crawfish Eco-Park, Jiangsu Province, China. We collected healthy, sexually matured male and female crawfish in September, October and November 2012, respectively. Before tissue collection, the male and female crawfish were cultured in a tank, and maintained in continuously aerated freshwater at ambient temperature (28±1°C) for 72 h. For the transcriptome experiments, the crawfish were placed in an ice bath for 1–2 min until anesthetized. Then the gonad tissues were removed through surgery, immediately frozen in liquid nitrogen, and separately stored at −80°C until further use. Tissues from three different individuals were taken every month, and the nine tissue samples were equally pooled as a single sample for RNA extraction. In total, testis and ovary samples were separately taken from nine male and nine female individuals. All gonad samples were taken from fully mature individuals and were expected to cover all stages of germ cell development.

### RNA extraction, cDNA synthesis, and sequencing

The total RNA of each testis or ovary sample were isolated using TRIzol Reagent (Invitrogen, USA) following the manufacturer’s instructions. The total RNA quality was determined using a NanoDrop (Thermo Scientific, USA), and the RNA integrity value (RIN) was checked using the RNA 6000 Pico LabChip of an Agilent 2100 Bioanalyzer (Agilent, USA). Total RNA of all samples was incubated with 10 U DNase I (Ambion, USA) at 37°C for 1 h, and purified with a MicroPloy (A) Purist Kit (Ambion, USA) following the manufacturer’s instructions. The purified mRNA was dissolved in RNA storage solution, and a NanoDrop (Thermo Scientific, USA) was used to determine the RNA final concentration.

Library construction and pyrosequencing was conducted by Beijing Genomics Institute (BGI; Shenzhen, China) on a 454 GS FLX system (Roche). The cDNA template for sequencing was generated using a cDNA library construction kit (Clontech, USA) according to the manufacturer’s protocol. The cDNA was fractionated into a range of 300–800 bp fragments. Specific adaptors were bound to the fragmented templates, and then used for purification, amplification, and sequencing steps. A half plate sequencing run was performed for each library on the 454 Genome Sequencer FLX instrument.

### Data assembly, clustering, and bioinformatics analysis

Through basic requirement, the original image data generated from the sequencing were transformed into sequence data, called raw data or raw reads. Subsequent analyses were based on clean reads obtained after a series of data processing steps. Assembly analysis of the transcriptome was carried out using Newbler v2.5 (Roche). Overlapping reads were first assembled into contigs. Contigs with connecting reads were compiled into isotigs. For further analysis, we first used BLASTx (E-value<10^−5^) to search the isotigs against various protein databases, including the National Center for Biotechnology Information (NCBI) non-redundant (nr) database, Swiss-Prot, Kyoto Encyclopedia of Genes and Genomes (KEGG), and Clusters of Orthologous Groups (COG). If the results of different databases were contradictory, a priority order of alignments from the nr, Swiss-Prot, KEGG, and COG databases was followed to decide the sequence direction.

### Sequence alignment and Phylogenetic analysis

Amino acid sequences related to *vasa* were downloaded from NCBI, sequence alignments were conducted with the BioEdit software [24] before construction of phylogenetic trees, and the result was imported to software MEGA5.0 [25]. Phylogenetic trees were generated using the neighbour-joining method and bootstrapped with 1000 iterations to evaluate the branch strength of the tree.

### In situ hybridazation

In situ hybridization studies were conducted to determine the exact location of *cyclin B* and *titin* mRNA expression in the gonad of *P. clarkii*. DIG-labeled RNA probes were synthesized using a DIG RNA labeling kit (Roche, Germany). Fragments of *cyclin B* and *titin* were amplified by PCR and cloned into PGEM-T easy vector, and the plasmid clones were used as templates to synthesize both sense and antisense RNA probes by in vitro transcription. The transcriptions were performed from 1 µg linearized plasmid using either T7 or SP6 RNA polymerases.

Testis and ovary tissue sections were dewaxed with xylene, hydrated in ethanol gradient. The technique used RNase-free reagents as described by Braissant [26]. Rehydrated tissues underwent the following procedures: wash in PBS with 0.1% active DEPC (2×15 min); equilibration in 5×SSC (15 min); prehybridization, 50% formamide, 5×SSC (30 min at 50°C); hybridization with 400 ng/mL of DIG-labeled probe, in 50% deionized formamide, 5×SSC, 1×Denhardt's solution, 10 mg/mL tRNA, 10% dextran sulfate (12–18 h at 55°C); washed in 2×SSC (30 min at room temperature); incubation in 2×SSC (1 h at 55°C); incubation in 0.1×SSC (1 h at 55°C); equilibration in buffer 1 (Tris 100 mM/NaCl 150 mM, pH 7.5) (5 min); incubation with anti-DIG antibody, AP-coupled, diluted 1∶1000 in buffer 2 [buffer 1 with 0.5% of Blocking Solution (Roche)] (2 h); washed in buffer 1 (2×15 min); equilibration in buffer 3 (Tris 100 mM/NaCl 100 mM, pH 9.5) (5 min); stained in buffer 3 containing 20 µl NBT/BCIP Stock Solution (Roche) (overnight); washed in running tap water (15 min); dehydrated in alcohol series; after which slides were mounted with cover slips.

### Identification of differentially expressed genes and quantitative real-time PCR analysis

The amount of isotig expression was calculated using the reads per kb per million reads method (RPKM), using the following formula:
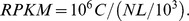



According to the method proposed by Audic [27], we developed a rigorous algorithm to screen genes that were differentially expressed between the two libraries. To judge the significance of differences in expressed genes, we used the FDR (false discovery rate) method [28] to determine the threshold of the *P*-value in multiple tests. In this study, we used three criteria: FDR≤0.001, the absolute value of the log_2_Ratio≥1, and a *P* value<0.01, as threshold to judge the significance of gene expression.

Within each of the categories for up-regulated and down-regulated genes in the ovary, we selected 14 genes at random for quantitative real-time PCR (RT-PCR) validation, and the specific primers (Table S1) were designed using the Primer Premier 5.0 program [29]. The total RNA of testis from 4 individuals and the total RNA of ovary from 4 individuals were reverse transcribed using 1 µg RNA of each tissue and the PrimeScript RT-PCR Kit (TaKaRa, Dalian, China) according to the manual. RT-PCR were performed using the SYBR premix Ex Taq Kit (TaKaRa, Dalian, China). The thermal profile was 95°C for 10 min, followed by 40 cycles of 95°C for 15 s, 58°C for 30 s, and 72°C for 30 s. The mRNA expression of each gene was quantified relative to β-actin (Forward, 5′-AAATCACGGCTCTGGCTCCCT-3′; Reverse, 5′-GAAGCATTTGCGGTGGACGAT-3′). The average cycle thresholds (CT) were used to determine fold-change. The relative quantification of gene expression was reported as a relative quantity (RQ) to the control value, and all experiments were performed with three technological replicates. The relative expression levels were calculated using the equation 2^−ΔΔCT^ (ΔCT = CT of target gene minus CT of β-actin, ΔΔCT = ΔCT of one gender sample minus ΔCT of opposite gender sample) [30].

### Data deposition

The sequences reported in this paper were deposited into the National Center for Biotechnology Information (NCBI) Sequence Read Archive with accession numbers SRP035515.

## Results and Discussion

### RNA sequencing and reads assembly

**Figure 1:**
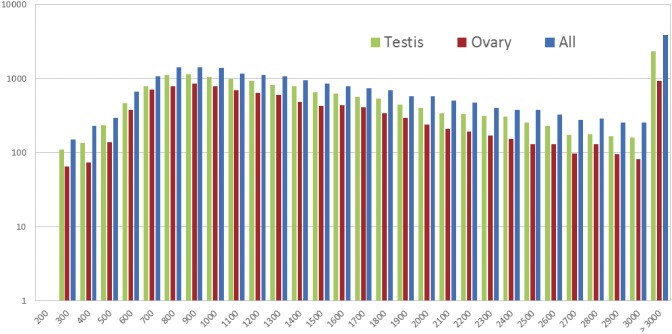
Assembly quality statistics of testis, ovary, and all isotigs from 454 sequencing. Length distribution of *de* *novo* assemblies of isotigs (x-axis indicates the sequence size (nt), y-axis indicates the number of assembled isotigs).

We conducted a 454 pyrosequencing for the testis and ovary cDNA libraries, resulting in 626,829 sequences with an average length of 775 bp and 508,144 sequences with an average length of 901 bp (Table 1), respectively. This produced a total of 943,840,902 nucleotides. Additionally, read lengths ranged from 55 to 1,780 bp in the testis library, and 53 to 1,780 bp in the ovary library. The assembly formed into 16,727 isotigs with an average length of 1827 bp in the testis library and 10,748 isotigs with an average length of 1544 bp in the ovary library. The length distribution of assembled isotigs is presented in Figure 1. The N50 isotig size of 2,255 bp in testis library was larger than that of 1,794 bp in ovary library. These assembly sequences represent the transcription and could be used for the further analysis.

**Table 1:** *P. clarkii* transcriptome assembly statistics.

Category	Testis	Ovary	All
Number of reads	626,829	508,144	1,134,993
Total base pairs (bp)	485,892,824	457,948,078	943,840,902
Average read length (bp)	775	901	832
Maximum read length (bp)	1,780	1,780	1,780
Minimum read length (bp)	55	53	53
Number of assembled isotigs	16,727	10,748	22,652
Average isotig length (bp)	1,827	1,544	1,921
Maximum isotig length (bp)	18,422	10,978	18,422
Isotig N50	2,255	1,794	2,474

### Functional annotation

**Figure 2:**
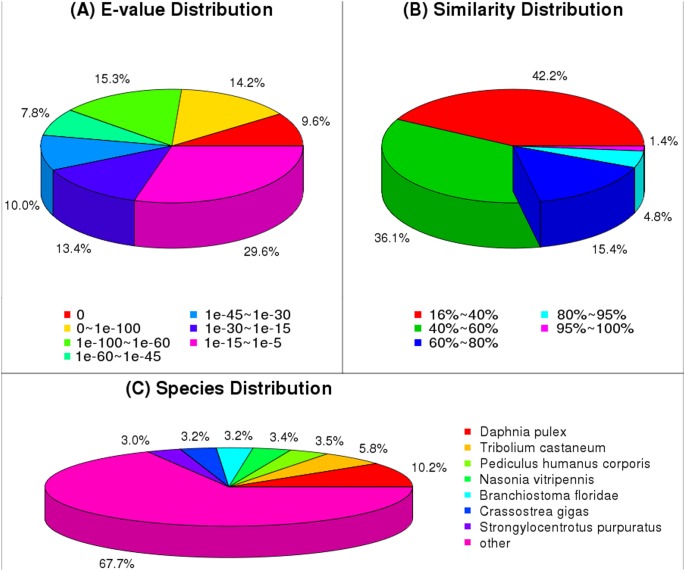
Characteristics of homology search of 454 sequences against nr database. (A) E-value distribution of BLAST hits for each unique sequence with a cut-off E-value of 1.0E-5. (B) Similarity distribution of the top BLAST hits for each sequence. (C) Species distribution shown as a percentage of total homologous sequences with E-value of ≥1.0E-5.

To understand the isotig functions, 22,652 isotigs were annotated using BLASTx alignment with an *E*-value smaller than 10E-5. A total of 15,667 (69.16%), 13,818 (61.00%), 12,419 (54.83%), 7,243 (31.98%), and 6,902 (30.47%) isotigs had significant matches with sequences in the NR, Swiss-Prot, KEGG, COG, and GO databases, respectively. Annotation results showed that many of the sequences have no homologous sequences in public databases due to the scarcity of species similar to *P. clarkii* in public databases. The *E*-value distribution of the top hits in the nr database showed that 39.1% of the mapped sequences had strong homology (less than 1.0E-60), whereas 60.9% of the homolog sequences ranged between 1.0E-5 and 1.0E-60 (Figure 2A). The similarity distribution showed a comparable pattern with 6.2% of the sequences having a similarity greater than 80%, while 93.8% of the sequences had a similarity ranging from 18% to 80% (Figure 2B). In terms of species distribution, similar isotigs were retrieved from *Daphnia pulex* (1,596, 10.19%), *Tribolium castaneum* (907, 5.79%), *Pediculus humanus corporis* (553, 3.53%), *Nasonia vitripennis* (530, 3.38%), *Branchiostoma floridae* (503, 3.21%), *Crassostrea gigas* (496, 3.17%), *Strongylocentrotus purpuratus* (473, 3.02%), and other species (10,608, 67.71%) (Figure 2C).

**Figure 3:**
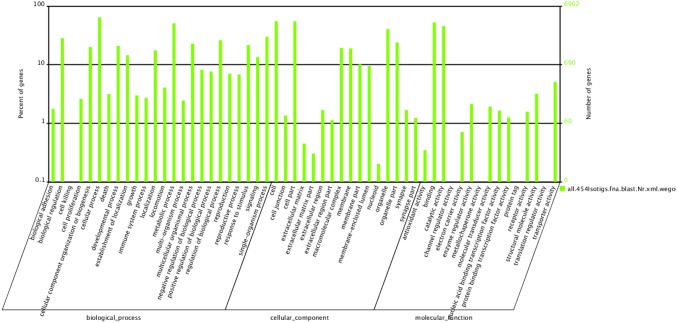
Histogram of GO classifications. Results summarized in three main categories: biological process, cellular component, and molecular function. Left y-axis indicates percentage of a specific category of genes in each main category. Right y-axis indicates number of genes in a category.

Gene ontology (GO) terms were used to classify the function of the predicted *P. clarkii* transcriptome. In total, 22,652 isotigs were analyzed with Blast2GO for GO classification [31] and distributed across 55 categories by WEGO [32] within the three ontologies ‘molecular function’, ‘cellular component’, and ‘biological processes’ (Figure 3). For the ontology ‘biological processes’, the most frequent categories were ‘cellular process’ (16.95%), ‘metabolic process’ (13.37%), ‘single-organism process’ (7.87%), and ‘biological regulation’ (7.46%), followed by ‘regulation of biological process’ (6.89%), ‘multicellular organismal process’ (6.02%), ‘response to stimulus’ (5.69%), ‘developmental process’ (5.52%), and ‘cellular component organization or biogenesis’ (5.25%). Other ‘biological process’ categories, such as ‘biological adhesion’ or ‘cell killing’ were present, but at lower percentages. In the ‘cellular component’ ontology, most of the terms were grouped into the ‘cell’ (22.94%) and ‘cell part’ (22.93%) categories, followed by ‘organelle’ (16.78%), ‘organelle part’ (9.94%), ‘macromolecular complex’ (8.01%), and ‘membrane’ (7.89%). Terms such as ‘cell junction’, ‘extracellular matrix’, ‘extracellular matrix part’, and ‘nucleoid’ were also present, but constituted a smaller proportion. The ‘molecular function’ ontology indicated that ‘binding’ and ‘catalytic activity’ contained over 84.52% of annotated sequences. Other categories, such as ‘translation regulator activity’, ‘channel regulator activity’, ‘metallochaperone activity’, and ‘protein tag’ were only present in small numbers.

The Kyoto encyclopedia of genes and genomes (KEGG) is a knowledge base for systematic analysis of gene functions in terms of the networks and molecules [33]. According to KEGG annotation information, a total of 12,419 isotigs were assigned to 256 basic metabolic pathways. The number of isotigs in different pathways ranged from 1 to 1,501 (Table S2), and the major pathway of ‘metabolic pathways’ included 12.9% of the isotigs. All pathway predictions are likely to be useful for future research focusing on their specific processes during gonadal development in *P. clarkii.*

### Sequence alignment and Phylogenetic analysis

In order to verify the accuracy of assembly, we observed sequences with a single complete open reading frame (ORF) as representative examples. Here we isolated the VASA sequences, for its critical functions in gonadal development. The sequence alignments and phylogenetic analysis results showed that *P. clarkii vasa* genes showed high identity with the homologues from other species, especially with that of crustaceans (Figure S1). It contained all nine conserved characteristic motifs, including AQTGSGKTAA, PTRELAVQ, GG, TPGR, DEAD, SAT, HGD, RGLD, and HRIGRTGR, similar to the other DEAD-box proteins. As shown in Figure S2, *vasa* of *P. clarkii* was remote from the vertebrate and mollusk *vasa* clade and clustered near the crustacean clade.

### Localization of cyclin B and titin transcripts in testes and ovaries

In different developing stages of testis and ovary, the expression of *cyclin B* and *titin* transcripts are presented in different distribution patterns. In ovary, the positive signals of these two genes were detected in some follicle cells surrounding nearly mature oocytes and nucleus (Fig. S3, B and G). In testis, *cyclin* and *titin* mRNA are present in spermatocytes and spermatogonia (Fig. S3 D and I). No hybridization signal exists in the negative control group (Fig. S3 C, E, H and J).

### Analysis of differentially expressed genes and verification using real-time PCR

**Figure 4:**
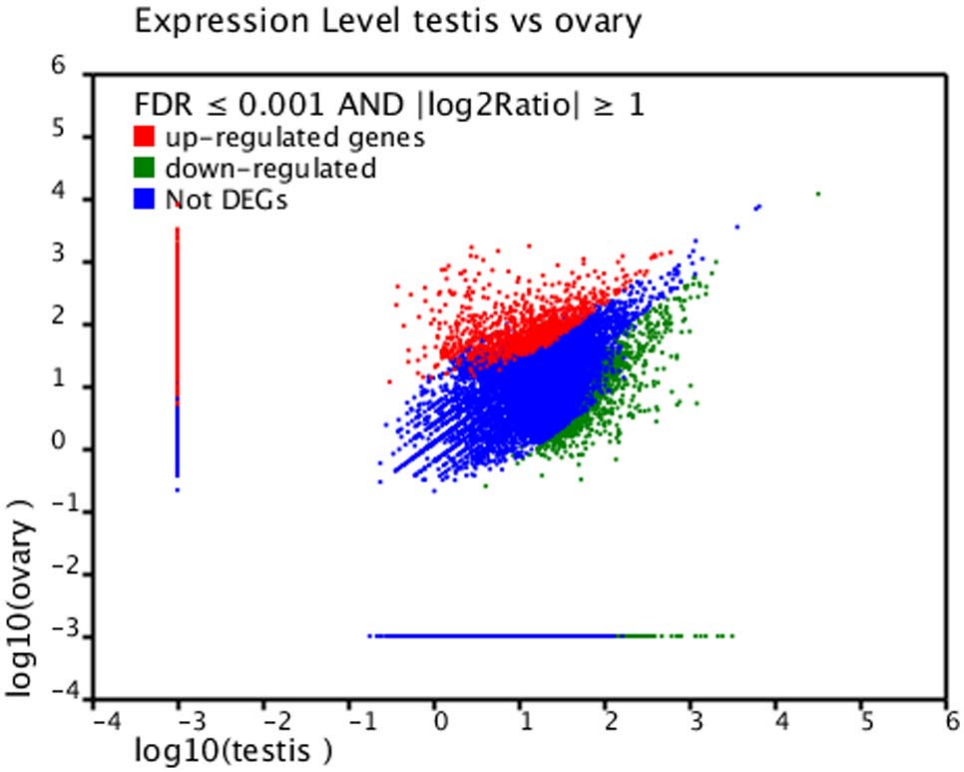
Scatter plots showing gene expression profiles in testis and ovary libraries of *P. clarkii*. Limits defined by FDR ≤0.001 and |log_2_ ratio| ≥1. Blue dots represent genes with similar expression in testis and ovary; red and green dots indicate up-regulated and down-regulated genes in ovary, respectively (>1.5-fold change, black diagonals).

In this study, statistical analysis of the differentially expressed genes between the testis and ovary libraries detected a list of 3,858 genes, of which 1,720 were up-regulated in the ovaries and 2,138 were down-regulated (Figure 4). These gonad specific up-regulated genes suggest their potential roles in gonadal development processes in *P. clarkii*. The most common GO terms associated with these features were ‘cell cycle regulation’ (36.8%), ‘muscle contraction and development’ (13.2%), and ‘transport’ (9.2%).

**Figure 5:**
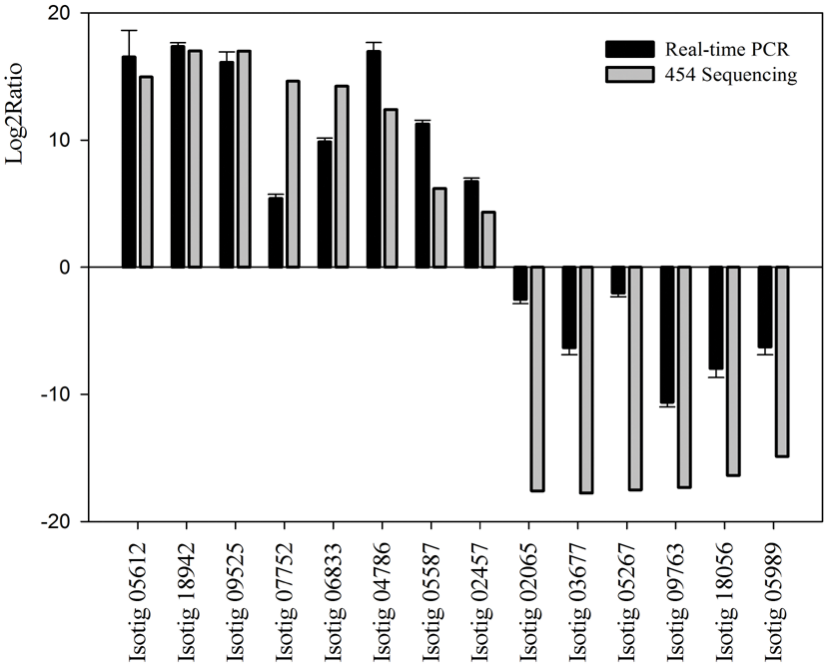
Histogram, comparison of gene expression between RT-PCR and 454 sequencing analysis. The RT-PCR are presented as the mean values of three repeats.

In order to ensure a reliable comparison between 454 sequencing and RT-PCR, we compared the mRNA levels of 14 candidate genes by RT-PCR. Of these 14 genes, eight were up-regulated and six were down-regulated in the ovary libraries. RT-PCR results verifying these differential expression profiles between testis and ovary libraries of *P. clarkii* are shown in Table S1 and Figure 5. Although the results of gene expression did not perfectly match to results detected by 454 sequencing, the up- and down-regulated trends were closely similar. Furthermore, it can be speculated that pooling of testis or ovary mRNAs from multiple individuals helped to level out potential variations. More genes will be validated in the future study.

### Putative regulatory genes involved in gonad development

It seems likely that the gonads contain more abundant transcription signals of gonadal development than its somatic tissue. We therefore constructed this database from testis and ovary libraries of *P. clarkii*. Based on the published literature and sequence annotation, several genes involved in gonad development were identified. These included ‘ovary development’, ‘ubiquitin proteasome pathway’, ‘cell cycle regulatory protein’ and ‘testis development’-related genes, such as *vitellogenin(Vg)*, *cyclin B*, *cyclin-dependent kinases 2*, *Dmc1* and *ubiquitin*. The genes in this list (Table 2) had been identified in previous studies to be essential for gonadal development.

**Table 2:** List of genes known to be involved in gonad development of *P. clarkii* transcriptome.

Description	Matched species	Size	*E* value
**Ovary development**			
*Vg*	*Homarus americanus*	9,510	0
*VgR*	*Macrobrachium rosenbergii*	7,460	2.00E-07
*cathepsin C*	*Marsupenaeus japonicas*	2,614	0
*cathepsin L*	*Penaeus monodon*	1,195	6.00E-104
*PL10-like protein*	*Macrobrachium nipponense*	5,465	0
*vasa-like protein*	*Marsupenaeus japonicas*	3,099	1.00E-172
*EF2*	*Eriocheir sinensis*	2,694	0
*EIF3*	*Pediculus humanus corporis*	5,538	0
*PTGR1*	*Penaeus monodon*	2,051	7.00E-107
**Cell cycle regulatory protein**			
*cvclin A*	*Penaeus monodon*	2,173	0
*cyclin B*	*Marsupenaeus japonicas*	2,595	1.00E-152
*CDK2*	*Macrobrachium rosenbergii*	1,461	3.00E-118
*CKS1*	*Acromyrmex echinatior*	2,456	2.00E-32
*CDC2*	*Penaeus monodon*	1,608	7.00E-168
*CKS1B*	*Scylla paramamosain*	2,307	1.00E-41
**Ubiquitin proteasome pathway**			
*ubiquitin*	*Papilio xuthus*	807	3.00E-104
*ubiquitin-conjugating enzyme E2 G1*	*Pediculus humanus corporis*	680	3.00E-88
*ubiquitin-conjugating enzyme E2 O*	*Camponotus floridanus*	1,872	4.00E-130
*UBR2*	*Camponotus floridanus*	7,476	2.00E-83
*hect E3 ubiquitin ligase*	*Pediculus humanus corporis*	10,308	0
*UBE4B*	*Clonorchis sinensis*	1,526	1.00E-08
*SUMO-1*	*Litopenaeus vannamei*	1,423	3.00E-46
**Testis development**			
*dynactin subunit 5*	*Penaeus monodon*	1,963	1.00E-99
*cyclophilin A*	*Eriocheir sinensis*	882	2.00E-83
*PCNA*	*Fenneropenaeus chinensis*	1,280	4.00E-135
*Nanos 2*	*Podocoryna carnea*	3,107	1.00E-14
*Nuclear autoantigenic sperm protein*	*Penaeus monodon*	2,188	2.00E-147
*Dmc1*	*Litopenaeus vannamei*	1,324	3.00E-175
*TEX14*	*Taeniopygia guttata*	4,874	1.00E-16
*Sperm-associated antigen*	*Pediculus humanus corporis*	2,015	0
*PRDM9*	*Bos taurus*	2,783	1.00E-26

Among these gonad-related genes, several ‘ovary development’ related genes were highly expressed in ovary tissues. As observed in many oviparous organisms, oocyte maturation depends on massive production of the egg yolk-precursor protein, *Vg*. *Vitellogenin receptor (VgR)* is involved in *vg* uptake by oocytes and plays a critical role in egg development [34]. The molecular characteristics of *Vg* and *VgR* have been described for many crustaceans [35]–[40]. In the current study, we found high occurrence of *Vg* and *VgR* in ovary of *P. clarkii*, and RT-PCR demonstrated that the expression level in ovary is significantly higher than in testis which provided some clues to further elucidate the function to ovary development of *P. clarkii* (Table S1). Cathepsins belong to a family of proteases that cleave other proteins and are ubiquitously present in almost all organisms [41], and involved in ovary maturation and embryo development [14], [42]. There are two kind of cathepsins: *cathepsin C* and *L* in our study, and were largely expressed in ovary libraries. Other ‘ovary development’ related genes including *elongation factor 2 (EF2)*, *prostaglandin reductase 1 (PTGR1)*, *eukaryotic translation initiation factor 3* (*EIF3*) have been found in ovary library, suggested the possible contribution of ovarian maturation [12], [43]–[44]. In this study, we found partial cDNAs from two members of the DEAD-box family, one belonging to the *vasa* subfamily and the other to the *PL10* subfamily. The *vasa* (and *vasa-like*) gene has been reported to play an essential role in germ cell development in higher metazoans, and localized in the oocyte cytoplasm throughout oogenesis [45]–[49]. Sequence and phylogenetic analysis results showed that *P. clarkii vasa* sequences present high identity with the homologues from other species, especially with that of crustaceans.

The cell cycle regulatory protein plays a key role in the oogenesis and oocyte development [10], [50]–[51]. The meiotic maturation of oocytes is regulated by the maturation promoting factor (MPF), a complex of *cyclin dependent kinases* (*CDK*), and *cyclin B*. In this project, a putative transcript of *cyclin B* and *cyclin dependent kinases regulatory subunit 1* (*CKS1*) were characterized with a higher expression levels in the ovary than testis of *P. clarkii*, and RT-PCR results are consistent with it. Further demonstrated the important role in ovarian development of *P. clarkii*. In situ hybridization revealed that *cyclin B* mRNA were clearly localized in the testis and ovary, these results suggested that *cyclin B* may play essential roles in the oogenesis and spermatogenesis of the crawfish (Table S3). The high expression of the *cyclin dependent kinases 2* (*CDK2*), *cell division cycle 2* (*CDC2*) and the *CDC28 protein kinase regulatory subunit 1B* (*CKS1B*) plays an important role in the ovary development and gametogenesis in crustaceans [11], [52]–[53]. In our study, similar to other reported animals, we found high expression of *CDK2*, *CDC2* and *CKS1B* in the *P. clarkii* testis and ovary libraries, show that it may be involved in the gametogenesis and gonad development of *P. clarkii*.

The ubiquitin proteasome pathway (UPP) is important for nearly every aspect of cellular life. It provides the cell with the ability to degrade proteins both specifically and temporally. The system generally includes three types of ubiquitin enzymes: ubiquitin-activating enzymes (E1s), ubiquitin-conjugating enzymes (E2s), and ubiquitin protein ligases (E3s). We found six kinds of ubiquitin enzymes in our current project, these ubiquitin-related homologous genes with different expression levels were found in the *P. clarkii* testis and ovary cDNA libraries, which suggests that ubiquitin proteins may play various roles in the reproductive process of *P. clarkii*. Recent studies have shown that UPP is involved in controlling the processes of gametogenesis, including meiosis control and reorganization of chromatin structure [54]. *Ubiquitin-conjugating enzyme E2 G1* and *ubiquitin-conjugating enzyme E2 O* have a highly conserved catalytic domain that is common to all E2s with a special carboxy-terminal extension different from other E2s. *E3 ubiquitin-protein ligase* (*UBR2*) localizes to meiotic chromatin regions and functions together with the *ubiquitin conjugating enzyme HR6B* in histone H2A ubiquitination during male meiosis [55]. *Hect E3 ubiquitin ligases* is involved in the ubiquitin-mediated degradation pathway. *Ubiquitin conjugation factor E4 B* (*UBR4B*) play an important role in cellular regulation [56]. *Small ubiquitin-related modifier1* (*SUMO1*) supports multiple roles in spermatogenesis [57]–[58].

Another interesting finding in the current study was the higher expression of genes in testis than ovary of *P. clarkii*, suggesting that involved in testicular development and spermatogenesis. *Cyclophilin A* and *dynactin subunit 5* were discovered functionally involved in testicular development of *P. monodon* [59]. The p*roliferating cell nuclear antigen* (*PCNA*) plays an important role in testis development, especially in the processes of mitosis and meiosis [55], and is involved in regulating spermatogenesis in the Japanese eel, *Anguilla japonica* [60]. *Nanos2* is expressed in self-renewing spermatogonial stem cells and maintains the stem cell state during murine spermatogenesis [61]. Deleting *Nanos2* may result in male sterility, owing to a loss of germ cells during fetal development [62]. The nuclear autoantigenic sperm protein provides the functional link between histones and cell cycle progression during meiosis [63]. The *testis expressed 14* (*TEX14*) may play a key role in regulating gene expression or modulating nuclear events during mammalian spermatogenesis [64]. *Dmc1* is a potentially useful indicator of the early stages of germ cell development [65], and plays a very important role during spermatogenesis. Research shows a possible association between *PR domain 9* (*PRDM9*) and azoospermia by meiotic arrest [66]. *Sperm associated antigen* is commonly expressed in male germ cells and plays an important role during spermatogenesis [67].

## Conclusions

The sequence data obtained in this study provide an archive for future research on gonadal development at a molecular level in *P. clarkii*. They could also serve as a theoretical foundation to solve problems in the breeding process. Here, we tried to elucidate their detailed role in the reproductive processes of *P. clarkii*. Future work will involve full-length amplification of candidate genes and validation of their functions.

## Supporting Information

Figure S1 **Alignment of the deduced amino acid sequences of** ***vasa*** **.** The amino acids conserved across all the eighteens species are shown in asterisks at the bottom. The GenBank accession numbers of the sequences are as follows: M. japonicus AEB00819; L. vannamei AAY89069; P. monodon AEB00820; F. chinensis ABQ00071; C. hawaiensis ACH92926; S. paramamosain ADR51551; M. nipponense ADB28894; P. carinicauda AGF90963.
(TIF)

Figure S2 **Neighbor-joining phylogenetic analysis of the** ***vasa*** **from** ***P. clarkii*** **.**
(TIF)

Figure S3 **Localization of** ***cyclin B*** **and** ***titin*** **transcripts in crawfish gonads.** The results of In situ hybridization with DIG-labeled antisense RNA probe (B and D for *cyclin B*; G and I for *titin*) and sense probe as negative control (C and E for *cyclin B*; H and J for *titin*) were shown. Regular histological section was stained with hematoxylin and eosin (A and F). O: oogonium; Cy: cytoplasm; N: nucleus; Nu, nucleolus; Fc: follicle cells; Sg: spermatogonium; Sc: Spermatocyte; St: Spermatid; The scale bar indicates 100 µm.
(TIF)

Table S1 **Real-time PCR confirmation of differential expressed genes.**
(DOCX)

Table S2 **KEGG biochemical pathways of** ***P. clarkii*** **isotig sequences.**
(XLSX)

Table S3 **List of the up-regulated and down-regulated genes between male and female libraries.**
(XLSX)
