# Supplementary figures and images for: Transcriptome Analysis of Red Swamp Crawfish Procambarus clarkii Reveals Genes Involved in Gonadal Development
Source: PLoS One. 2014 Aug 13;9(8):e105122. doi: 10.1371/journal.pone.0105122 (PMC4132113; doi:10.1371/journal.pone.0105122)

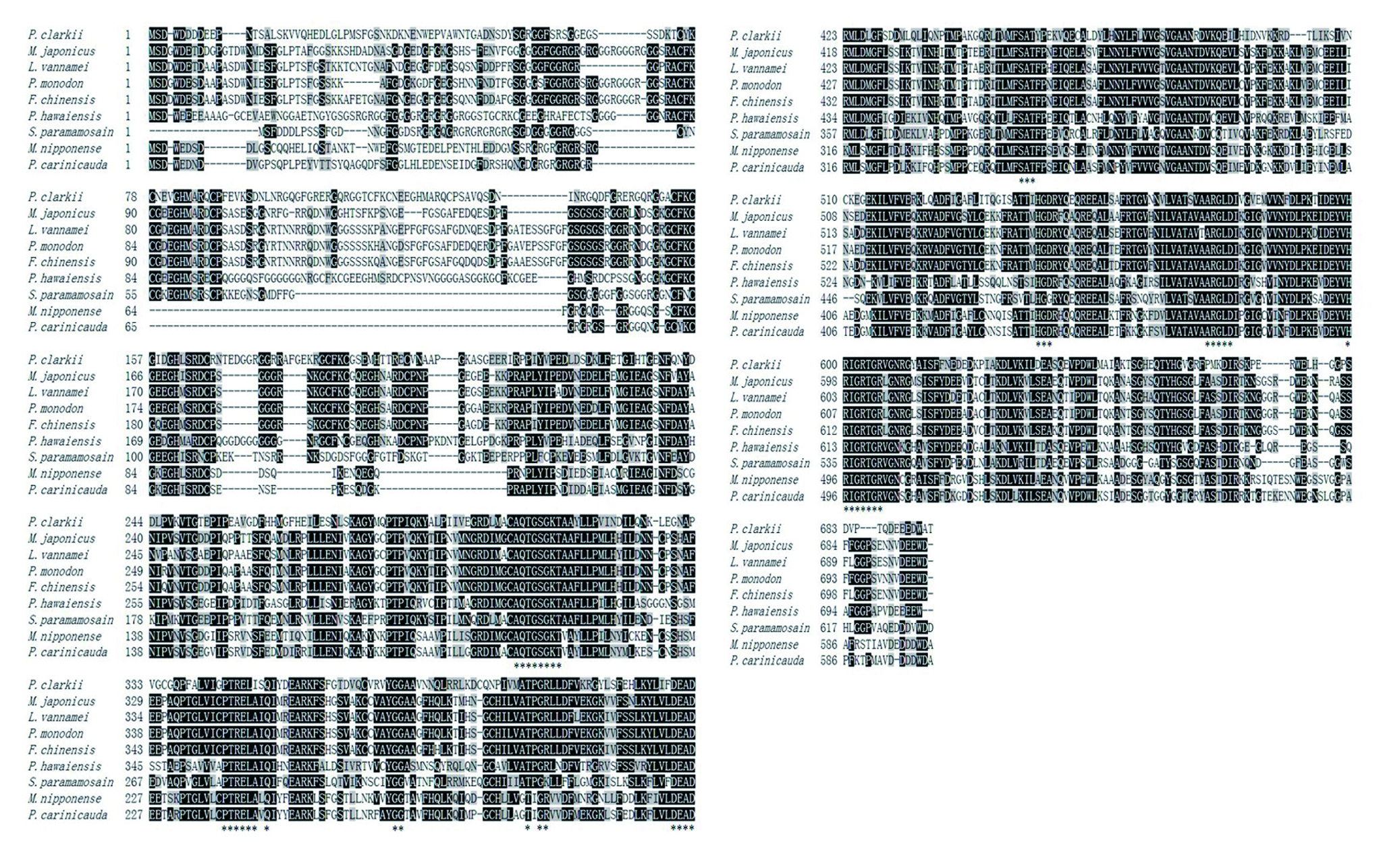

Supplement: Figure S1 — Alignment of the deduced amino acid sequences of vasa . The amino acids conserved across all the eighteens species are shown in asterisks at the bottom. The GenBank accession numbers of the sequences are as follows: M. japonicus AEB00819; L. vannamei AAY89069; P. monodon AEB00820; F. chinensis ABQ00071; C. hawaiensis ACH92926; S. paramamosain ADR51551; M. nipponense ADB28894; P. carinicauda AGF90963. (TIF) [file pone.0105122.s001.tif]

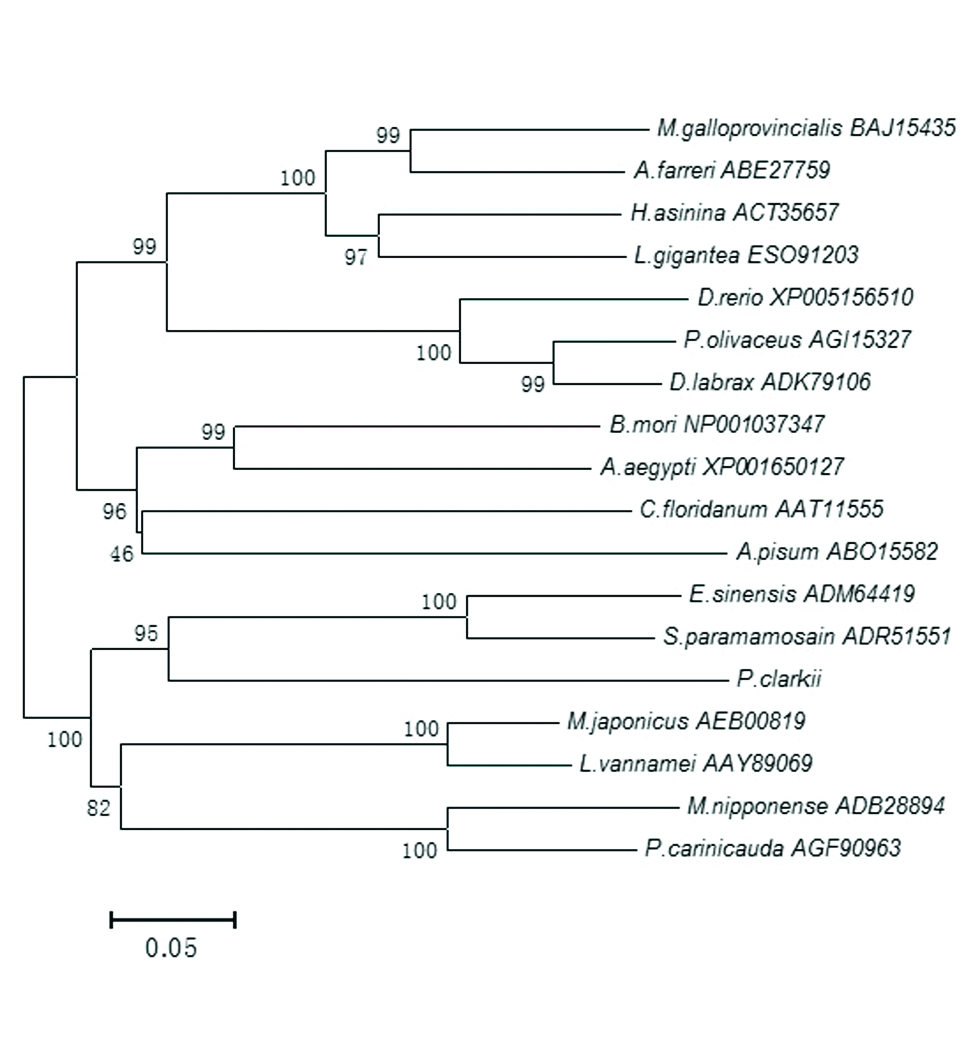

Supplement: Figure S2 — Neighbor-joining phylogenetic analysis of the vasa from P. clarkii . (TIF) [file pone.0105122.s002.tif]

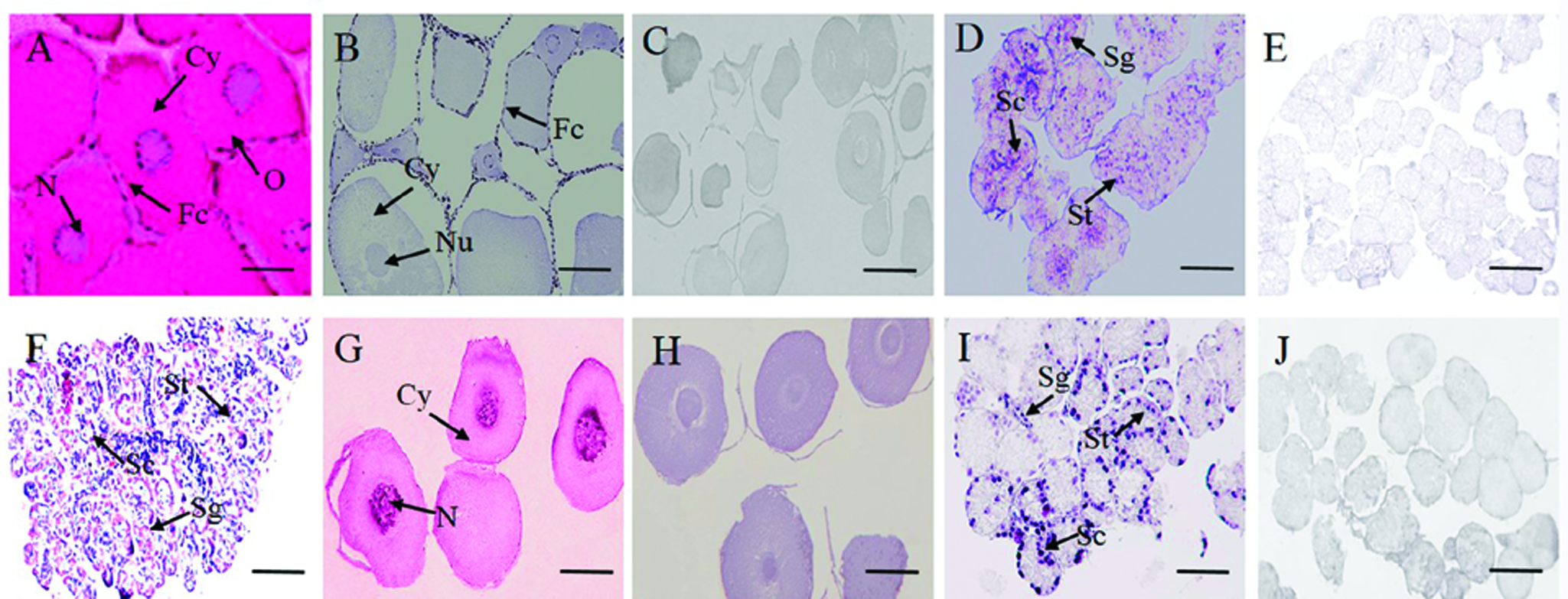

Supplement: Figure S3 — Localization of cyclin B and titin transcripts in crawfish gonads. The results of In situ hybridization with DIG-labeled antisense RNA probe (B and D for cyclin B; G and I for titin) and sense probe as negative control (C and E for cyclin B; H and J for titin) were shown. Regular histological section was stained with hematoxylin and eosin (A and F). O: oogonium; Cy: cytoplasm; N: nucleus; Nu, nucleolus; Fc: follicle cells; Sg: spermatogonium; Sc: Spermatocyte; St: Spermatid; The scale bar indicates 100 µm. (TIF) [file pone.0105122.s003.tif]
