# Supplementary material for: Transcriptome Analysis of Red Swamp Crawfish Procambarus clarkii Reveals Genes Involved in Gonadal Development
Source: PLoS One. 2014 Aug 13;9(8):e105122. doi: 10.1371/journal.pone.0105122 (PMC4132113; doi:10.1371/journal.pone.0105122)
Supplement: Table S1 — Real-time PCR confirmation of differential expressed genes. (DOCX) [file pone.0105122.s004.docx]

**Table S1. Real-time PCR confirmation of differential expressed genes**

| **Isotig ID** | **Nr-annotation** | **Primer** | **PCR product length** | **454 sequencing**  **log2 (ovary/testis)** | **Real-time PCR**  **log2 (ovary/testis)** |
| --- | --- | --- | --- | --- | --- |
| Isotig 05612 | cyclin B* | F: TCCCCATGAACCGTCACAT  R: CCTGAAGAACGCTACCACCA | 210 | 14.96 | 16.54 |
| Isotig 18942 | mannose-binding protein | F: TTGAGTGTTGGTGTAAGTTTGGGTC  R: TGGGAGGGTGTTCTGAATCTGT | 153 | 17.01 | 17.36 |
| Isotig 09525 | C-type lectin 6 | F: CAGCCAATATGTCACTATGCCG  R: GGTAAACTGTCCTGCTCCAACTC | 135 | 17.00 | 16.12 |
| Isotig 07752 | tripsin, putative | F: TGCTCCATCCTCTGTCCGT  R: CCCCAACTCAAGTGGTCCTT | 129 | 14.63 | 5.41 |
| Isotig 06833 | ABC transporter | F: ACAGGCGGTTTATGAGTTCCC  R: TGGTGTCTTTCTGGTTGGTGC | 170 | 14.24 | 9.86 |
| Isotig 04786 | vitellogenin receptor* | F: CCGTGTCACCCATCAAAGTC  R: TGGCAGTGGACGAGATAAAGAC | 150 | 12.40 | 16.96 |
| Isotig 05587 | vitellogenin* | F: ATCACGCTCAACGGCATCTT  R: CTCAGAATCCAACGACCAATCA | 175 | 6.19 | 11.27 |
| Isotig 02457 | Cyclin-dependent kinases regulatory subunit 1* | F: AGTGTTAGAAGCCCCAGAATGC  R: GGTGAATGGTGAGGGGTAGG | 150 | 4.33 | 6.76 |
| Isotig 02065 | Titin | F: ACGTGGATCTCATCCTTAACTTCT  R: ACCTTGCCCTGTGACCTGTAC | 88 | - 17.59 | - 2.52 |
| Isotig 03677 | C-type lectin | F: CACGCCAACCACCAACTATC  R: TACCACAACGAACCCAACAAC | 159 | - 17.76 | - 6.34 |
| Isotig 05267 | histidine rich protein 2 | F: ACAGCTCTTCGGATTCGTAGTC  R: TGAACAGAGGCATCAGGGTAAA | 100 | - 17.51 | - 2.02 |
| Isotig 09763 | merozoite surface protein 1 | F: TCCGTCCATCTCGACTGTGA  R: CGACCTCGCCTTACCTAATCT | 142 | - 17.32 | - 10.63 |
| Isotig 18056 | mucin-2 precursor | F: TGGGAGGCGAAGGCAATCT  R: CGTGTTGGTGCATGAAGGGTAC | 134 | - 16.38 | - 7.97 |
| Isotig 05989 | sporozoite surface protein | F: GCTGCGGAAGTTGAGGGAGT  R: AGTCAGACCAGCCATCCACATA | 100 | - 14.88 | - 6.28 |
